# A multi-omic analysis reveals a predictive value of tertiary lymphoid structures in improving the prognosis of colorectal cancer patients with BRAF mutation

**DOI:** 10.3389/fimmu.2025.1662573

**Published:** 2025-09-01

**Authors:** Chao Qin, Shumin Cheng, Jingyun Ma, Lujing Li, Yun Leng, Lei Zheng, Huiying Chen, Hui Mo, Shi Li, Yuhong Liang, Yi Zhang, Wenxia Li, Jing Liang, Yuxuan Liu, Junxuan Mai, Linlin Hou, Di Wang, Ke Zhu, Bihui Huang

**Affiliations:** ^1^ Scientific Research Center, The Seventh Affiliated Hospital of Sun Yat-Sen University, Shenzhen, China; ^2^ Department of Pathology, Sun Yat-Sen Memorial Hospital, Sun Yat-Sen University, Guangzhou, China; ^3^ Department of Ultrasound, The Seventh Affiliated Hospital, Sun Yat-Sen University, Shenzhen, China; ^4^ Department of Aesothology, The Seventh Affiliated Hospital of Sun Yat-Sen University, Shenzhen, China; ^5^ Guangdong Province Key Laboratory of Malignant Tumor Epigenetics and Gene Regulation, Sun Yat-Sen Memorial Hospital, Sun Yat-Sen University, Guangzhou, China; ^6^ School of Pharmacy, Macau University of Science and Technology, Macao, Macao SAR, China; ^7^ Department of Thoracics, The Seventh Affiliated Hospital, Sun Yat-Sen University, Shenzhen, China; ^8^ Department of Pathology, The Seventh Affiliated Hospital of Sun Yat-Sen University, Shenzhen, China; ^9^ School of Medicine, Sun Yat-Sen University, Shenzhen, China; ^10^ Health Management Center, The Seventh Affiliated Hospital of Sun Yat-Sen University, Shenzhen, China

**Keywords:** colorectal cancer, BRAFmutation, tertiary lymphoid structures, tumor microenvironment, prognosis

## Abstract

**Background:**

*BRAF* mutations are prevalent in colorectal cancer (CRC) and generally confer a poor prognosis. Tertiary lymphoid structures (TLS), a critical component of the tumor immune microenvironment, exist in various malignancies and often correlate with improved immunotherapy response and survival. However, whether TLS can counteract the adverse prognostic effects of *BRAF* mutations in CRC remains unexplored. This study characterizes TLS features (location, number, maturity) as well as correlation to the *BRAF* mutation status and clinicopathological characteristics in CRC, and specifically evaluates the potential role of TLS in mitigating the negative prognostic impact of *BRAF* mutations.

**Methods:**

Single-cell RNA sequencing data from GSE146771, GSE146771, GSE200997, GSE205506, and GSE231559, along with bulk RNA-seq data from the TCGA CRC cohort, were analyzed. Prognostic genes were identified using univariate Cox regression and least absolute shrinkage and selection operator (LASSO) regression, and subsequently used to construct TLS-related prognostic signatures. Kaplan-Meier survival analysis and receiver operating characteristic (ROC) curve analysis were used to evaluate the predictive performance of the signature. Immune infiltration was assessed using the ESTIMATE and CIBERSORT algorithms. Histopathological evaluation of TLS was conducted in tissue sections from 200 CRC patients. Clinicopathological features were compared between the *BRAF* wild-type (BRAF^WT^) and *BRAF* mutant (BRAF^MT^) groups. Associations between BRAF mutation status and TLS location, number, maturity, as well as overall survival (OS), were analyzed.

**Results:**

TLS displayed distinct expression patterns within the CRC tumor microenvironment. A 10-gene prognostic model was developed based on LASSO regression analysis. Patients with BRAF^MT^ CRC exhibited unfavorable clinicopathological characteristics, including poor differentiation, advanced T stage, and lymph node metastasis. Meanwhile, BRAF^WT^ CRC was associated with a greater number and higher maturity of TLS. Notably, patients with BRAF^WT^, TLS-high (TLS^High)^, and BRAF^WT^-TLS^High^ subgroups showed significantly improved OS compared to other groups.

**Conclusion:**

TLS-related prognostic signatures serve as effective tools for predicting CRC outcomes. Moreover, intratumorally TLS may enhance the prognosis of patients with BRAF^MT^ CRC, highlighting its potential as a therapeutic and prognostic biomarker. Colorectal cancer, *BRAF* mutation, tertiary lymphoid structures, tumor microenvironment, prognosis.

## 1 Introduction

Colorectal cancer (CRC) is one of the most common malignant tumors worldwide, and the incidence of new CRC cases is projected to reach 3.15 million by 2040 (1, 2). Currently, the treatments for CRC patients mainly include surgery, chemotherapy, immune check point inhibitors and targeted therapy (3, 4). Anti-EGFR antibody is now recommended as first-line therapy for patients possessing wild-type oncogenes of the RAS-MAPK pathway, such as KRAS, NRAS, and/or BRAF (5). About 8%-15% of CRC patients carry BRAF mutations, with BRAF^V600E^ being the most common subtype (6–8). Despite considerable advances in CRC treatment, efficient management of advanced-stage CRC, particularly in patients carrying BRAF mutations, remains a major challenge.

The BRAF oncogene encodes BRAF protein, which is localized downstream of RAS, leading to the activation of the mitogen-activated protein kinase (MAPK) pathway. Several hot spots were identified in BRAF mutations, with V600E (a substitution of valine by glutamic acid at codon 600) accounting for up to 80% of all BRAF mutations (9, 10). It was reported that CRC patients with BRAF^V600E^ mutation have a poorer prognosis and require more intensive chemotherapy or combination therapy with targeted drugs (11–13).

The tumor microenvironment (TME) is the “soil” for tumor genesis, progression, and metastasis. TME usually includes immune cells, fibroblasts, blood vessels, and stromal components (14). Tertiary lymphoid structure (TLS) is an acquired ectopic organized immune cell aggregation structure in non-lymphoid organs, which usually occurs in chronic inflammatory diseases, such as autoimmune diseases, infectious diseases, and tumors. TLSs are usually composed of B-cell follicles with germinal centers, dendritic cells, hyperendothelial venules, and T-cell zones, and are spontaneously formed by lymphocytes at inflammatory sites (15, 16). As an important part of the immune microenvironment, TLS is closely associated with immunotherapy efficacy and cancer patient prognosis (17). Recent studies have demonstrated a positive prognostic correlation between TLS and various tumors, including non-small cell lung cancer (18), melanoma (19), sarcoma (20), breast cancer (21), and prostate cancer (22).

To date, there are few studies on the association between BRAF gene mutation and TLS signatures in CRC (23). In this study, we analyzed single-cell RNA sequencing (scRNA-seq) data of CRC to explore the expression patterns of TLSs across different cell types. Based on these data, we developed a new TLS-associated prognostic signature. Furthermore, we divided patients into high-risk and low-risk groups according to the score of the prognostic signature, and then explored the contrast of *BRAF* mutation status and other biological characteristics between the two groups. Our findings reveal a potential connection between TLS signatures and *BRAF* mutations in CRC, providing new insights into CRC prognosis and treatment strategies.

## 2 Materials and methods

### 2.1 Dataset source and preprocessing

The RNA expression data of colorectal cancer, including clinical information, were obtained from the Cancer Genome Atlas Program (TCGA) database (https://portal.gdc.cancer.gov/). The scRNA-Seq sequencing data sets GSE146771 (n  = 20), GSE166555 (n  = 13), GSE200997 (n  = 16), GSE205506 (n  = 27), and GSE231559 (n  = 6) were obtained from the Gene Expression Omnibus (GEO) database (https://www.ncbi.nlm.nih.gov/), and all tumor samples were included in the analysis. The 121 TLS-related gene set was employed from the integration of published articles (19, 24–30).

### 2.2 Unsupervised clustering of TLS

We used the “ConsensusClusterPlus” package (31) to classify patients into different molecular subtypes by unsupervised clustering techniques based on the expression matrix and clinical information of the TCGA colorectal cancer cohort. The optimal number of clusters (k = 2) was determined by the consensus matrix, cumulative distribution function (CDF), and relative changes in the area under the CDF curve. After clustering, the cluster assignments were validated by t-distributed stochastic neighbor embedding (t-SNE). The survival associated with each molecular subtype was evaluated by Kaplan-Meier survival analysis.

### 2.3 Construction and validation of TLS prognostic signature

To establish a TLS-related prognostic signature, we subjected a 121-relevant-gene set to univariate Cox proportional hazards regression analysis to screen genes associated with survival (P< 0.05). Subsequently, LASSO regression (10-fold cross-validation, “glmnet” package) was performed to select the optimal penalty parameter λ according to the 1-SE (standard error) criterion to minimize the prediction error. Finally, the gene set was streamlined to ten core genes (*CCL19, CCL22, ICOS, IGHG1, JCHAIN, CD37, XBP1, FCMR, TNFRSF13C*, and *FCRLA*). The risk score of each patient was calculated based on the expression levels of these genes and weighted by their corresponding LASSO coefficients as follows:


$$Risk\;Score\;=\;\sum \left(Expression_{i}\times Coefficient_{i}\right).$$

According to the median risk score, patients were divided into high-risk and low-risk groups. The overall survival between the two risk groups was compared using Kaplan-Meier survival analysis. The predictive accuracy of the prognostic model at 1, 3, and 5 years was evaluated using time-dependent receiver operating characteristic (ROC) analysis with the area under the curve (AUC) as an indicator. In addition, we compared the risk grouping with other clinical indicators to demonstrate the reliability of the prognostic signature. A nomogram model based on clinicopathological features and prognostic scores was constructed using the “rms” R package.

### 2.4 Differential expression and functional enrichment between clusters

Differentially expressed genes (DEGs) between two molecular clusters based on TLS-related gene sets were identified using “limma” (|log2FC| > 1, adjusted P< 0.05). Functional enrichment analysis of these DEGs, including Gene Ontology (GO) and Kyoto Encyclopedia of Genes and Genomes (KEGG), was performed using the R package “clusterprofiler” (version 4.2.2) (32)to explore the biological functions of the DEGs.

### 2.5 Tumor microenvironment characterization of clusters

To investigate the differences in tumor microenvironment between TLS-based molecular clusters, immune cell infiltration was quantified using the CIBERSORT deconvolution algorithm. The signature matrix of 22 immune cell types (LM22) was used as a reference; 1,000 permutations were performed, and quantile normalization was enabled (perm = 1000, QN = TRUE). The normalized gene expression data (after log transformation) were input as a mixing matrix. Spearman correlation analysis was performed to explore the potential association between risk score and immune infiltrating cells. In addition, the ESTIMATE score (stromal/immune score) and Tumor Immune Dysfunction and Exclusion (TIDE) score (http://tide.dfci.harvard.edu/) were evaluated to assess differences in immunogenicity.

### 2.6 Analysis of genetic variation in different TLS risk groups in COAD

The mutation data and clinical information of the COAD dataset in MAF format were downloaded from the TCGA database using the R package TCGAbiolinks, and the R package maftools was used to compare the differences in mutation spectra between TLS^High^ and TLS^Low^ subgroups, including mutation burden (TMB), high-frequency mutation genes, and mutation patterns, to evaluate the impact of TLS status on the genomic characteristics of patients with *BRAF* mutations.

### 2.7 Gene set enrichment analysis

To explore the potential biological mechanism of TLS risk grouping in BRAF-mutated colorectal cancer, we used the R package “clusterProfiler” to perform gene set enrichment analysis (GSEA). DESeq2 was used to perform differential analysis between TLS^High^ and TLS^Low^ groups in *BRAF* mutation samples, and the screening criteria were genes with p-value< 0.05 and |log_2_FoldChange| > 1. The gene set used for enrichment analysis was from the HALLMARK collection of the MSigDB database (https://www.gsea-msigdb.org/gsea/msigdb) in the “msigdbr” package.

### 2.8 Drug sensitivity analysis

We used the R package “oncoPredict” to perform drug sensitivity prediction analysis. First, we obtained the standardized cell line expression matrix and the corresponding drug response (IC50) data from the GDSC2 database (https://www.cancerrxgene.org/) as the training set. Then, the transcriptome expression data of our TCGA-COAD cohort were formatted and aligned with the gene names before being input into the model. The “calcPhenotype()” function was used to predict the sensitivity of the samples to multiple drugs included in GDSC2. During the prediction process, the Empirical Bayes method was used to correct the batch effect (batchCorrect = “eb”), and removeLowVaryingGenes = 0.2 was set to remove low-variance genes to optimize model performance. Finally, the predicted drug IC50 values ​​were integrated with clinical information and visually compared in different risk groups (TLS^High^ and TLS^Low^) to evaluate the potential response differences between the two groups to different drugs.

### 2.9 Patients and specimens

200 CRC patients were enrolled, who primarily underwent their first surgical resection at Sun Yat-sen Memorial Hospital, and were pathologically diagnosed with CRC from 2016 to 2018. The study was approved by the Ethics Committee of Sun Yat-sen Memorial Hospital (SYSKY - 2024-098-01), and all patients recruited in the study signed informed consent.

### 2.10 Immunohistochemical staining

IHC was performed to analyze the mutation of the BRAF protein (V600E) in all CRC cases. CD3, CD20, and CD21 were performed to analyze the TLS maturity. The sections used for IHC were obtained from paraffin-embedded tissues of CRC patients and stained. Briefly, paraffin-embedded tissues were cut into 4μm sections and were first dewaxed with xylene 3 times, followed by gradient rehydration with different concentrations of ethanol, followed by epitope repair by boiling the sections in citrate buffer (pH 6.0) or Tris-EDTA (pH 9.0) under high pressure for 10 min. The sections were treated with 3% hydrogen peroxide for 15min to eliminate endogenous peroxidase and then blocked with 5% bovine serum albumin (BSA) for 30min. The sections were incubated with primary antibody overnight in a chamber at 4°C. On the second day, the slides were taken out and washed with PBS 3 times, 5 minutes each time, and then incubated with HRP-conjugated secondary antibody at 37°C for 30 minutes. Finally, the slides were washed with PBS 3 times, then incubated with DAB, and followed by counterstaining using hematoxylin.

### 2.11 Hematoxylin and eosin

H&E staining was performed to identify TLS characteristics in all CRC cases. The sections used for H&E staining were obtained from paraffin-embedded tissues of CRC patients, stained, and evaluated by two pathologists. Paraffin-embedded sections were dewaxed and rehydrated as described in “immunohistochemical staining (IHC)”, then stained with hematoxylin, rinsed with running tap water, re-stained with eosin, and finally dehydrated and sealed.

### 2.12 BRAF mutational status and TLS quantification

The whole section was scanned by OLYMPUS BX53 (Olympus, JAPAN) and double-blind evaluated by two independent pathologists. IHC staining was used to verify the mutation status of *BRAF*, and H&E staining was used to reveal the presence of TLS in CRC. TLS can be divided into 3 types according to the different anatomical subregions (intra-tumor, invasive margin, and peri-tumor). According to their maturity, TLSs can be classified as lymphoid aggregates (Agg) and lymphoid follicles (Fol); Fol can be further subdivided into Fol-I (lymphoid follicles without germinal centers) and Fol-II (with germinal centers).

The TLS scoring system was established based on the abundance of TLSs in different subregions. TLS abundance was divided into 4 groups: (a) score 0 indicates no TLS, which is equivalent to TLS negative CRC; (b) score 1 represents 1 – 5 TLSs; (c) score 2 represents 6 – 10 TLSs; (d) score 3 represents over 10 TLSs.

### 2.13 Single-cell RNA analysis

For each scRNA-seq dataset (GSE146771, GSE166555, GSE200997, GSE205506, and GSE231559), we retained genes detected in at least three cells and excluded cells with mitochondrial gene content exceeding 10%. Cells with fewer than 300 or more than 5,000 detected genes were also removed. After quality control, the five datasets were integrated, and batch effects were mitigated using the “Harmony” algorithm (33). Cell types were manually annotated based on canonical marker gene expression profiles. Cell-cell communication was inferred using the “CellChat” package (34), which utilizes a curated ligand-receptor interaction database. In addition, the “AddModuleScore” function was applied to score each cell for a predefined TLS-related gene signature.

### 2.14 Statistical analysis

GraphPad Prism software (version 9.3.2) and SPSS software V.23.0 (IBM) were used to perform statistical analysis. The chi-square test is used to compare counted data. The overall survival (OS) time was defined as the time in months from operation to death. Kaplan-Meier was used to plot survival curves, and progression-free survival was compared using the Log-rank test. A two-tailed p-value less than 0.05 was considered statistically significant.

## 3 Results

### 3.1 Construction of COAD prognostic risk score model based on TLS-related gene sets

We first analyzed the expression profiles of 121 TLS-related genes (**Supplementary Table 1**) in tumor tissues and adjacent normal tissues from the TCGA-COAD cohort. Differential expression analysis revealed that 82.6% of TLS-related genes (100 genes) were significantly dysregulated in tumor tissues (**Supplementary Figure 1A**). To further investigate TLS-related transcriptional patterns, we applied unsupervised clustering to 442 tumor samples with complete clinical data using the R package ConsensusClusterPlus. Based on the empirical cumulative distribution function (CDF) curves, k = 2 was selected as the optimal number of clusters, providing the highest intra-cluster correlation and lowest inter-cluster correlation (**Supplementary Figures 1B-D**). Two different TLS-related gene expression patterns, designated as TLS-related clusters 1 and 2, were observed. t-SNE analysis demonstrated clear separation between the two clusters (**Supplementary Figure 1E**), supporting the reliability of the clustering. Kaplan-Meier (KM) survival analysis showed that the patients in cluster 2 had significantly shorter overall survival (OS) compared to those in cluster 1 (**Supplementary Figure 1F**). These findings suggest that TLS-related gene expression patterns are closely associated with the CRC prognosis and may serve as a potential indicator for patient stratification.

**Figure 1:**
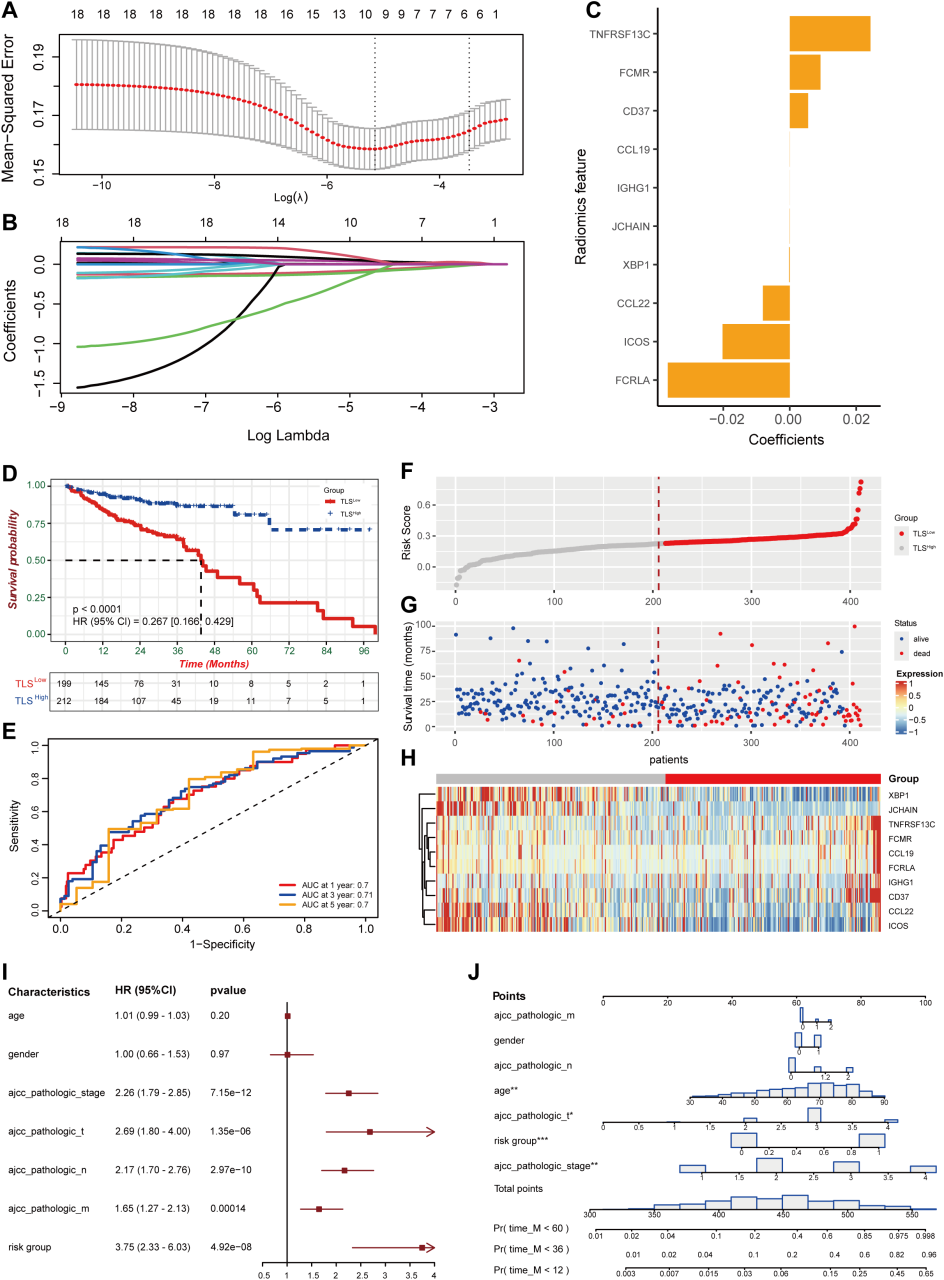
Construction of prognostic features for colorectal cancer based on TLS-related genes. **(A)** Coefficient profile of TLS-related gene sets. **(B)** Determination of the optimal parameter (Lambda) in LASSO. **(C)** Construction of a risk model with the determined 10 genes. **(D)** Kaplan-Meier analysis of survival rate. **(E)** ROC curve analysis of 1-year, 3-year, and 5-year survival rates of the risk model. **(F, G)** Ranking and scatter plots of prognostic score distribution and patient survival status. **(H)** Heat map of 10 OS-related gene expressions. **(I)** Forestplotter displays the hazard ratio of each clinical indicator. **(J)** Nomogram for predicting 1-year, 3-year, and 5-year survival rates of colorectal cancer.

To further study the expression pattern of TLS-related genes associated with CRC prognosis, we first performed univariate Cox regression analysis on the 121 TLS-related genes and identified 18 genes with significant prognostic value (p< 0.05, **Table 1**). Using LASSO penalized Cox regression analysis, we refined these to 10 key prognostic genes (*CCL19, CCL22, ICOS, IGHG1, JCHAIN, CD37, XBP1, FCMR, TNFRSF13C*, and *FCRLA*) based on the minimum criterion penalty parameter (**Figures 1A-C**). The prognostic score was calculated based on the expression levels of these genes, and the formula was as follows:

**Table 1:** Univariate Cox regression analysis of TLS-related genes in TCGA-COAD.

Gene	HR	L95CI	H95CI	pvalue
CCL19	1.00589247	1.00206682	1.00973273	0.00251145
CCL22	0.95057001	0.90675333	0.99650402	0.03525527
ICOS	0.87576979	0.77273276	0.99254588	0.0377904
MS4A1	1.0315384	1.00605165	1.05767082	0.01498931
CD19	1.07853845	1.03461233	1.12432953	0.0003654
CD79B	1.05767187	1.01030279	1.1072619	0.01646626
CXCR5	4.39559267	1.38351826	13.9652908	0.01206023
IGHG1	1.00005747	1.0000041	1.00011083	0.03480252
JCHAIN	0.99944999	0.99904272	0.99985742	0.00815264
XBP1	0.99849552	0.99702143	0.99997179	0.04578355
CD22	1.05410256	1.01537389	1.09430842	0.00580126
CD37	1.02506493	1.00160806	1.04907114	0.03608188
FCMR	1.04182798	1.0156864	1.0686424	0.00157544
TNFRSF13C	1.11473158	1.04576756	1.1882435	0.00085794
FCRLA	1.08703668	1.00686991	1.17358631	0.03275098
NIBAN3	1.2202592	1.07341334	1.38719397	0.00234327
RASGRP2	1.10636989	1.03657976	1.1808588	0.0023607
TCL1A	1.04051128	1.00710998	1.07502035	0.01705325

Risk score = 2.59e-02*TNFRSF13C + 9.61e-03*FCMR + 5.67e-03*CD37 + 3.25e-05*CCL19 + 3.50e-06*IGHG1 - 4.43e-05*JCHAIN –2.13e-04*XBP1 - 8.25e-03*CCL22 - 2.07e-02*ICOS - 4.00e-02*FCRLA.

Based on the median risk score, we divided the dataset into high-risk (TLS^Low^) and low-risk (TLS^High^) groups. Patients in the TLS^Low^ group exhibited significantly shorter OS than those in the TLS^High^ group (**Figure 1D**). The prognostic model demonstrated high stability and accuracy, with the ROC curve (AUC) of 0.7 for 1-year survival prediction, 0.71 for 3-year survival prediction, and 0.7 for 5-year survival prediction (**Figure 1E**). Consistent with this, patients in the high-risk score group exhibited a higher probability of death as shown in the survival distribution diagram (**Figure 1F, G**). The heat map further illustrated the association between the 10 genes and the prognostic scores (**Figure 1H**). The forest plot combined with clinical parameters and the TLS risk model showed that the risk group had a higher hazard ratio (**Figure 1I**). Finally, a nomogram was constructed to predict the overall survival of the TCGA-COAD cohort (**Figure 1J**).

### 3.2 Immune profiles of colorectal cancer patients with divergent prognostic outcomes

**Figure 2:**
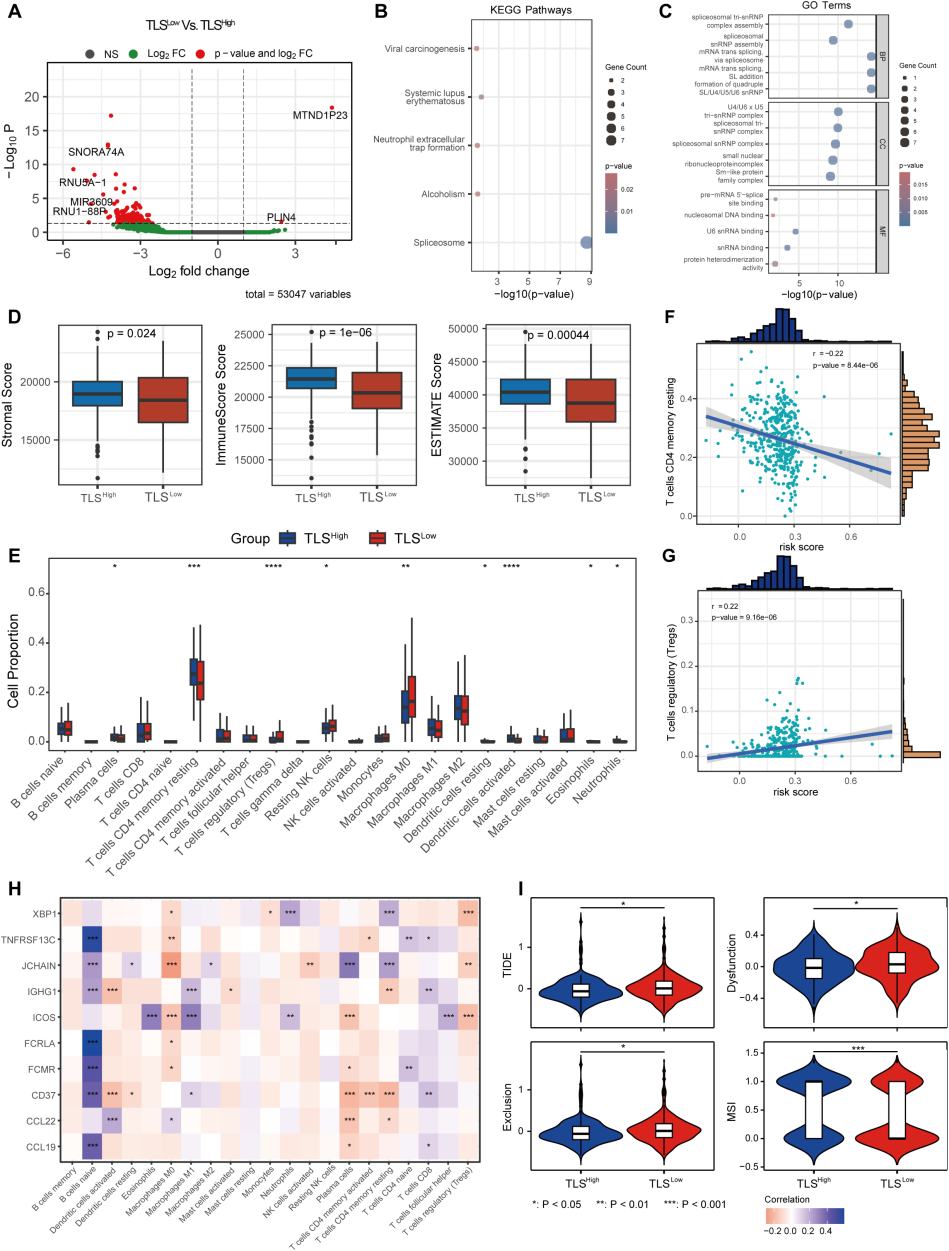
Immune microenvironment and TMB of COAD with different prognostic scores. **(A)** Volcano plot of differentially expressed genes between the two groups with different prognostic scores. KEGG **(B)** and GO **(C)** enrichment analysis of DEGs between the two groups. **(D)** ESTIMATE score between the two risk groups. **(E)** CIBERSORT immune cell infiltration ratio between the low-risk and high-risk groups. Correlation between the changes in the ratio of T cells CD4 memory resting cells **(F)** and T cells regulatory (Tregs) cells **(G)** and risk score. **(H)** Correlation between prognostic score gene expression and immune cells. **(I)** TIDE score between the two risk groups.

To explore the different biological behaviors of each TLS cluster, we performed differential analysis using the limma package and identified 174 DEGs associated with the TLS clusters (**Figure 2A**). GO enrichment analysis results revealed that these differential genes were significantly enriched in biological processes and molecular functions such as RNA splicing and complex assembly, nucleosome structure and nuclear RNA binding activity, and protein-protein interactions (**Figure 2B**). KEGG pathway analysis further indicated that these differential genes were mainly enriched in viral oncogenic pathways and immune-related response pathways (**Figure 2C**).

We next evaluated the infiltration of stromal cells and immune cells in the TCGA-COAD cohort using the ESTIMATE algorithm. The results revealed that the immune score, stromal score, and ESTIMATE score were significantly lower in the TLS^Low^ group compared to the TLS^High^ group (**Figure 2D**), indicating a lower level of immune and stromal cell infiltration in TME and suggesting an immunologically “cold” phenotype. To further characterize the immune landscape, we applied the CIBERSORT algorithm to assess the composition of immune cells in each sample. The TLS^Low^ group showed significantly higher proportions of regulatory T cells (Tregs), resting NK cells, and M0 macrophages compared to the TLS^high^ group, suggesting an immunosuppressive state. In contrast, the TLS^High^ group showed significantly increased proportions of plasma, CD4 memory resting T, DC resting, eosinophils, and neutrophils, indicating a more active immune state (**Figure 2E**). Moreover, we observed a distinct correlation between the risk score and immune cell composition (**Supplementary Figure 2**). The proportion of T cells CD4 memory resting cells decreased with a correlation coefficient of -0.22 (*p* = 8.44e-06) (**Figure 2F**), while the proportion of Tregs increased with a correlation coefficient of 0.22 (*p* = 9.16e-06) (**Figure 2G**). In addition, the expression of genes included in the prognostic score model was significantly associated with the abundance of various immune cell types (**Figure 2H**).

TIDE analysis showed that the TLS^Low^ group exhibited significantly higher total TIDE score, Exclusion (immune rejection score), and Dysfunction (immune dysfunction score) compared to the TLS^High^ group, while the MSI score was significantly lower. These findings indicate that tumor cells in the TLS^Low^ CRC group have a stronger immune escape ability than those in the TLS^high^ group. Moreover, the elevated TIDE scores and reduced MSI scores in TLS^Low^ patients suggest a poorer response to immune checkpoint inhibitor (ICI) treatment (**Figure 2I**).

### 3.3 TLS can improve the prognosis of patients with BRAF mutations in COAD

**Figure 3:**
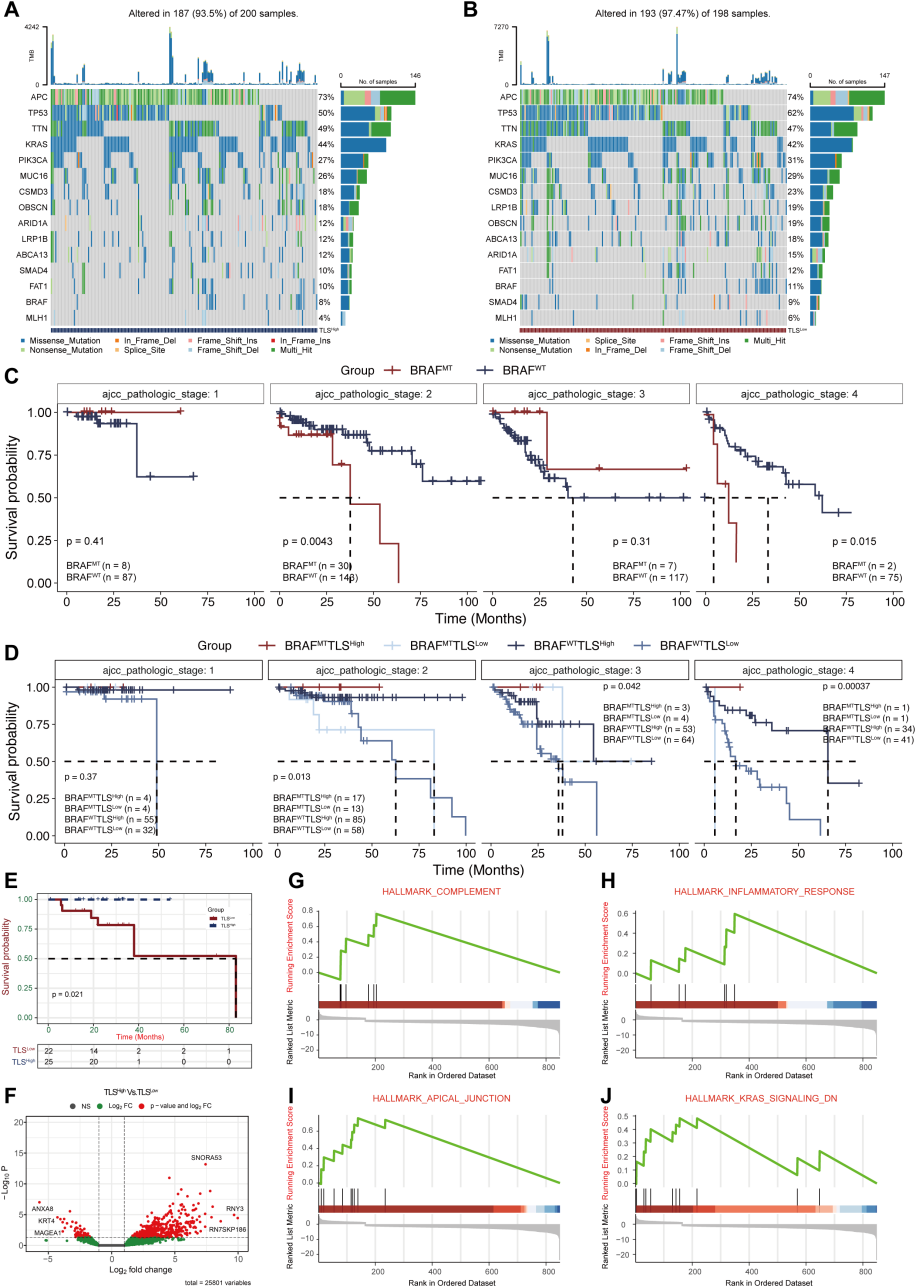
Analysis of BRAF mutations in COAD patients with different prognostic scores. **(A, B)** Mutation profiles between the TLS^High^ and TLS^Low^ groups. **(C)** Survival analysis of BRAF mutations (MT) and wild-type (WT) at different AJCC pathological stages. **(D)** Survival analysis of BRAF mutations combined with TLS^High/Low^ groups at different AJCC pathological stages. Survival **(E)** and DEGs analysis **(F)** of TLS groups within BRAF mutation samples. **(G-J.)**GSEA enrichment analysis of DEGs.

In colorectal cancer, about 10%-20% of patients carry BRAF mutations, which are usually associated with strong invasiveness and poor prognosis (7). One aim of our study is to investigate whether TLS could offset the adverse effects of BRAF mutations. In the TCGA-COAD cohort, we observed that the frequency of BRAF mutations was significantly lower in the TLS^High^ group compared to the TLS^Low^ group (8% vs. 11%, **Figures 3A, B**), suggesting a potential biological relevance between TLS and BRAF status. Stratified survival analysis by AJCC pathological stage revealed stage-specific patterns: BRAF mutated type (BRAF^MT^) TLS^High^ patients were significantly higher than BRAF wild type (BRAF^WT)^ TLS^Low^ patients in both stage II and stage IV (**Figures 3C, D**). In addition, there were significant differences in gender and age (Chi-square test, **Supplementary Figures 6B,C**). This stage-specific benefit implies that TLS may selectively ameliorate BRAF mutation-driven pathology, which provides a theoretical basis for implementing immune-centric treatment strategies in BRAF mutation subgroups.

We further conducted survival analysis in BRAF^MT^ samples from the TCGA dataset, which confirmed that the TLS^High^ patients showed significantly better survival than their TLS^Low^ counterparts (**Figure 3E**). Differential expression analysis identified 613 DEGs between the two groups (**Figure 3F**). GSEA analysis (**Supplementary Table 2**) revealed that these DEGs were significantly enriched in pathways such as “HALLMARK COMPLEMENT” (NES = 2.27, p=1.09e-03), “HALLMARK INFLAMMATORY RESPONSE” (NES = 1.76, p=2.74e-02), “HALLMARK APICAL JUNCTION” (NES = 2.80, p=3.48e-05), and “HALLMARK KRAS SIGNALING DN” (NES = 1.87, p=1.35e-02). These results suggest that TLS may offset the adverse prognosis caused by BRAF mutation by activating the anti-tumor immune microenvironment and inhibiting oncogenic signaling pathways.

### 3.4 Prognostic significance of BRAF mutation and TLS in CRC tissues

A total of 200 CRC patients were enrolled, among whom 40 (20%) harbored the *BRAF^V600E^* mutation (**Supplementary Figure 3A**). Clinicopathologic features, including sex, age (≤ or >50), degree of tumor differentiation (poor, moderate, well), T stage, and lymphatic metastasis, were analyzed (**Supplementary Figures 3B-F**). The CRC patients ranged in age from 25 to 85 years, with a mean of 57 years, and a male-to-female ratio of 1.5:1. Pathological analysis revealed that 42 cases (21%) were well-differentiated, 135 (68%) moderately differentiated, and 23 (11%) poorly differentiated. According to the AJCC TNM staging system (8th Ed), 24 patients (12%) were classified as T1 stage, 74 (37%) as T2 stage, 73 (37%) as T3 stage, and 29 (14%) as T4 stage. Among them, 94 patients were found with lymph node metastasis. Statistical analysis showed that the BRAF^V600E^ mutation was significantly correlated with tumor differentiation, T stage, and lymph node metastasis. The characteristics of the patients are summarized in **Supplementary Table 3**.

**Figure 4:**
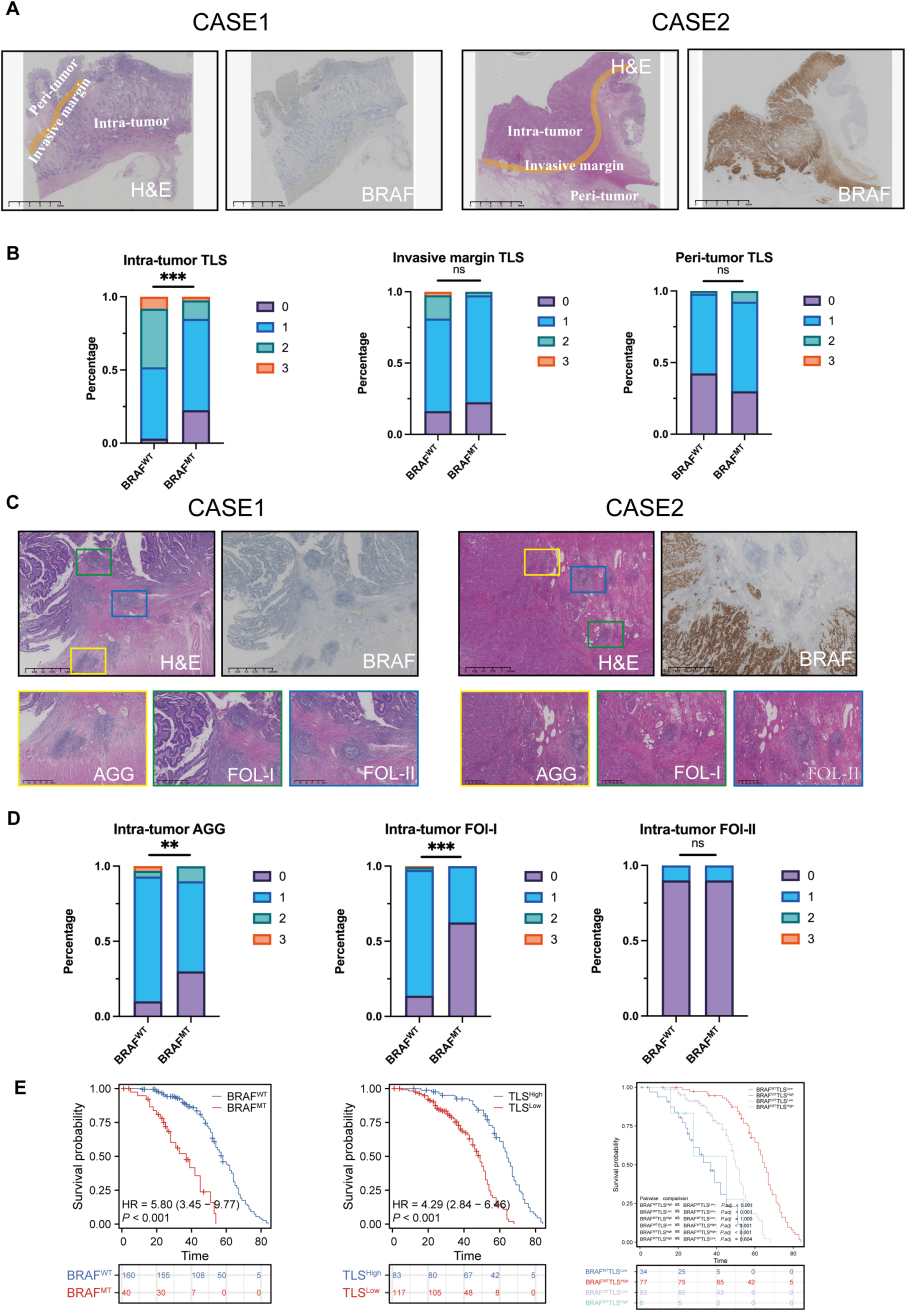
Intra-tumoral TLS with greater quantity and maturity in colorectal cancer may counteract the poor prognosis caused by BRAF mutations. **(A)** H&E and IHC staining of two CRC patients, showing BRAF status and TLS in different subregions, intra-tumor, invasive margin, and peri-tumor. **(B)** Percentage of TLS numbers between BRAF^WT^ and BRAF^MT^ CRCs at intra-tumor, invasive margin, and peri-tumor subregions. ns: no statistically significant difference. **(C)** H&E and IHC staining of two CRC patients, showing TLS with different maturity, AGG, FOL-I, and FOL-II TLS. **(D)** Statistical analysis of intra-tumor AGG, Fol-I, and FOL-II TLS between BRAF^WT^ and BRAF^MT^ CRCs. **(E)** Analysis of the probability of survival between BRAF^WT^ and BRAF^MT^ CRC patients, TLS^High^ and TLS^Low^ CRC patients. BRAF^WT^-TLS^High^, BRAF^WT^-TLS^Low^, BRAF^MT^-TLS^High,^ and BRAF^MT^-TLS^Low^ CRC patients. TLS abundance was divided into 4 groups: score 0 indicates no TLS, score 1 represents 1 – 5 TLSs, score 2 represents 6 – 10 TLSs, and score 3 represents over 10 TLSs. ns, no statistically significant difference; **p<0.01; ***p<0.001.

We analyzed the distribution, quantity, and maturity of TLS in CRC and their correlation with BRAF mutational status. There were 64 (40%) and 13 (8%) patients who were classified as grade 2 and grade 3 intra-tumor TLS in BRAF^WT^ CRC, while there were only 5 (13%) and 1 (3%) in BRAF^MT^ CRC. These findings indicate a significant correlation between BRAF mutational status and the abundance of the intra-tumor TLSs. However, no significant differences were observed between BRAF mutational status and TLS grade in the invasive margin or peri-tumor (**Figures 4A, B**).

Next, we examined the correlation between the maturity of intra-tumor TLS and BRAF mutational status. We found that 16 (10%) of BRAF^WT^ CRCs and 12 (30%) of BRAF^MT^ CRCs had zero intra-tumor AGG TLS. Among BRAF^WT^ cades, 138 (86%) patients had FOL-I TLS, which were further classified into grades 1, 2, and 3. In contrast, only 15 (38%) patients with BRAF^MT^ CRCs harbored FOL-I TLS, and none were categorized as grade 2 or 3. These findings indicate that intra-tumoral AGG and FOL-I are significantly correlated with BRAF mutation status, whereas FOL-II showed no significant correlation (**Figures 4C, D**). The statistics are shown in **Supplementary Tables 4**, **Supplementary Tables 5**.

Based on BRAF mutational status, CRC patients were classified into BRAF^WT^ and BRAF^MT^ groups. According to the median number of intra-tumoral TLS, they were further stratified into TLS^High^ (>5) and TLS^Low^ (≤5) groups. Kaplan-Meier survival analysis revealed significant differences in OS between BRAF^MT^ and BRAF^WT^ (*P<0.0001*) and between TLS^High^ and TLS^Low^ (*P<0.0001*, **Figures 4D, E**). Subsequently, these patients were categorized into four groups based on both BRAF mutational status and intra-tumor TLS (BRAF^WT^TLS^High^, BRAF^WT^TLS^Low^, BRAF^MT^TLS^High,^ and BRAF^MT^TLS^Low^). Prognostic analysis showed that patients in the BRAF^WT^TLS^High^ group had the most favorable survival outcome, with a median survival of 65 months (*P<0.0001*, **Figure 4E**).

### 3.5 TLS profiles in colorectal cancer scRNA-seq datasets

To validate and extend our findings from the TCGA-COAD cohort, we further investigated the single-cell RNA sequencing (scRNA-seq) datasets of GSE146771, GSE166555, GSE200997, GSE205506, and GSE231559. Following quality, control integration, and removal of batch effects, a total of 113,379 cells were retained for further analysis (**Supplementary Figure 4A**). Dimensionality reduction and clustering analysis identified 14 cell lineages, including CD4 memory T cells, CD8 T cells, Plasma, Tregs, Epithelial tumor cells, activated B, Macro_Mono, Endothelial Cell, CAFs, T cells, B cells, MEP, Memory B cells, and DC cells (**Supplementary Figure 4B**). The number and proportion of these cell types varied across datasets (**Supplementary Figures 4C, D**). The heat map shows the top five marker genes for each cell type (**Supplementary Figure 4E**).

**Figure 5:**
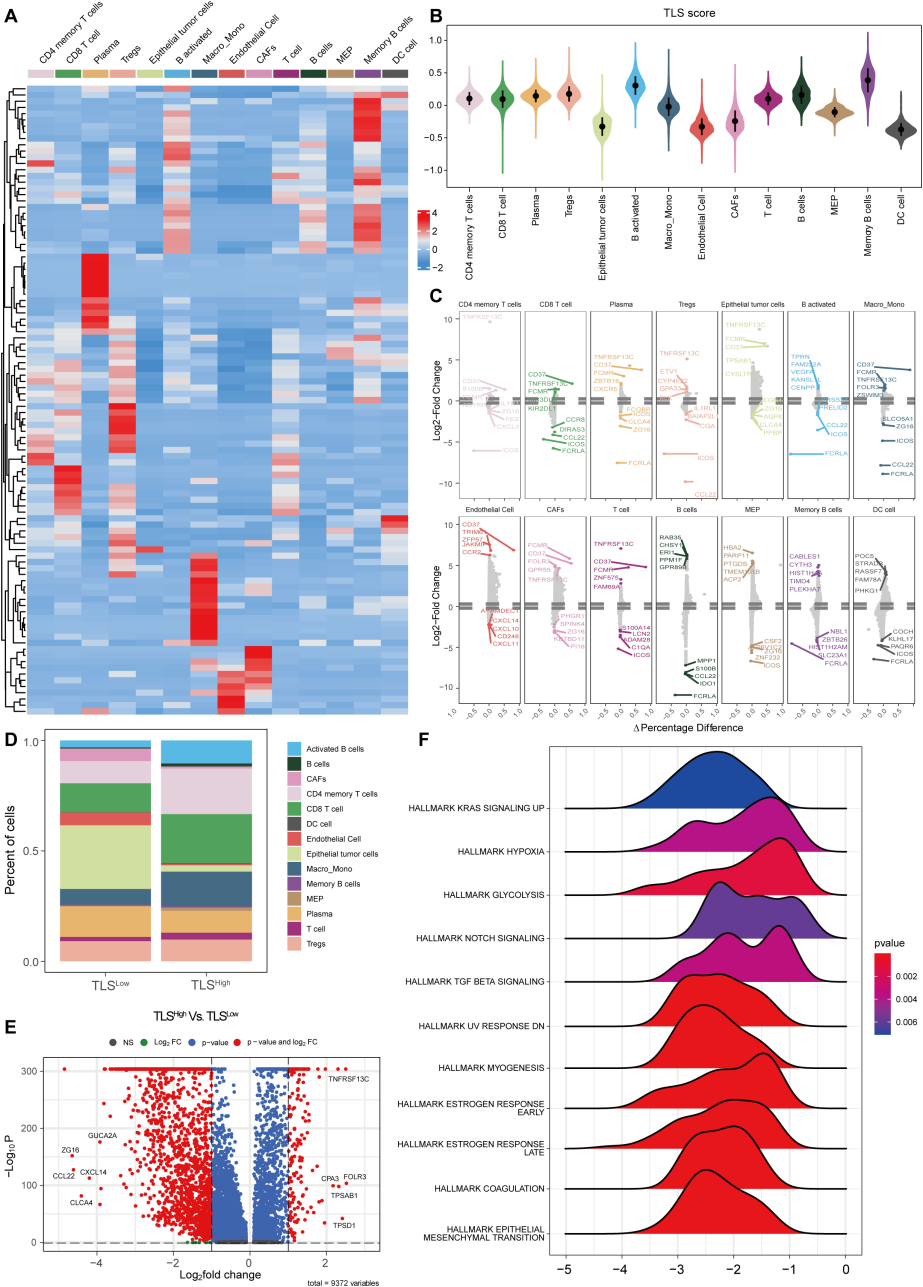
Single-cell analysis of TLS in colorectal cancer. **(A)** Heat map of TLS-related gene set expression in each cell type. **(B)** Violin plot of TLS scores in each cell type. **(C)** Differential expression analysis of TLS^High^ and low groups in each cell type. **(D)** Histogram of cell proportions in different TLS groups. **(E)** Volcano plot of differential expression analysis in different TLS groups. **(F)** GSEA ridge plot of different TLS groups.

Single-cell sequencing data revealed different expression patterns of TLS-related genes in different cell types (**Figure 5A**). We used the AddModuleScore algorithm to merge all 121 TLS genes into a gene set and calculated the TLS score for each cell type. Consistent with the TCGA-COAD cohort, activated B cells and memory B cells showed the highest TLS scores, while tumor cells and stromal cells (e.g., CAFs) had lower scores (**Figure 5B**). To explore the functional differences associated with TLS status, based on the risk score model constructed by the TCGA-COAD cohort, we stratified single cells into TLS^High^ and TLSLow groups and identified differentially expressed genes (DEGs) between the two groups in each cell subtype. Differential expression analysis showed that in TLS^High^ tumors, various immune cells and structural cells exhibited distinct pro-inflammatory and immune activation properties. Specifically, CD4 memory T cells highly expressed TNF, while CD8 T cells upregulated TNF, IFNG, GZMK, PDCD1, and CTLA4, indicating that T cells were in an activated but partially exhausted state. Activated B cells and memory B cells significantly upregulated CD80, CD86, and CD40, reflecting their enhanced antigen presentation and co-stimulatory capabilities. Macrophage/monocyte TNF, IL1β, and IL6 expression increased, suggesting an enhanced proinflammatory response. Endothelial cells upregulated ICAM1 and VCAM1, which may promote immune cell recruitment and angiogenesis (**Figure 5C**). These results are very similar to those observed in bulk RNA sequencing data (e.g., enhanced immune infiltration, antigen presentation, and T cell activation in TLS^High^ tumors), thus strengthening the consistency between multi-omics datasets. In summary, the TLS^High^ state is achieved not only by immune cell enrichment but also by enhanced antigen presentation, T cell activation, and proinflammatory responses, which provides a basis for the improved prognosis of patients with BRAF mutant COAD.

To further explore the differences in immune microenvironment and functional pathways between TLS^High^ and TLS^Low^ groups, we stratified the samples accordingly. The analysis revealed different cell type compositions. The proportions of CD4 memory T cells, CD8 T cells, activated B cells, Memory B cells, and Macro_Mono in the TLS^High^ group were significantly increased, while the proportions of epithelial tumor cells and CAF were notably decreased (**Figure 5D**). Differential gene expression analysis between the two groups is shown in the volcano plot (**Figure 5E**). GSEA enrichment analysis indicated that the TLS^Low^ group was enriched in multiple pathways related to immunosuppression and tumor progression (**Figure 5F**), including “HALLMARK KRAS SIGNALING UP”, “HALLMARK HYPOXIA”, “HALLMARK GLYCOLYSIS”, “HALLMARK TGF BETA SIGNALING”, and “HALLMARK NOTCH SIGNALING”. These results are consistent with those obtained in our previous GSEA analysis in the TCGA-COAD cohort (**Figure 3J**), indicating that the TLS^Low^ group may exhibit stronger immune escape and metabolic reprogramming compared to the TLS^high^ group. In addition, enrichment of “HALLMARK EPITHELIAL MESENCHYMAL TRANSITION” and “HALLMARK COAGULATION” pathways in the TLS^Low^ group implies stronger invasiveness and angiogenesis compared to the TLS^high^ group.

Collectively, our scRNA-seq analyses independently confirmed the bulk RNA-seq findings from the TCGA-COAD cohort, demonstrating that TLSHigh tumors are characterized by enhanced immune activation, antigen presentation, and improved prognostic features. These consistent observations across bulk and single-cell levels underscore the robustness and clinical relevance of TLS-related gene expression patterns in colorectal cancer.

### 3.6 TLS regulates the microenvironment by enhancing cellular communication

**Figure 6:**
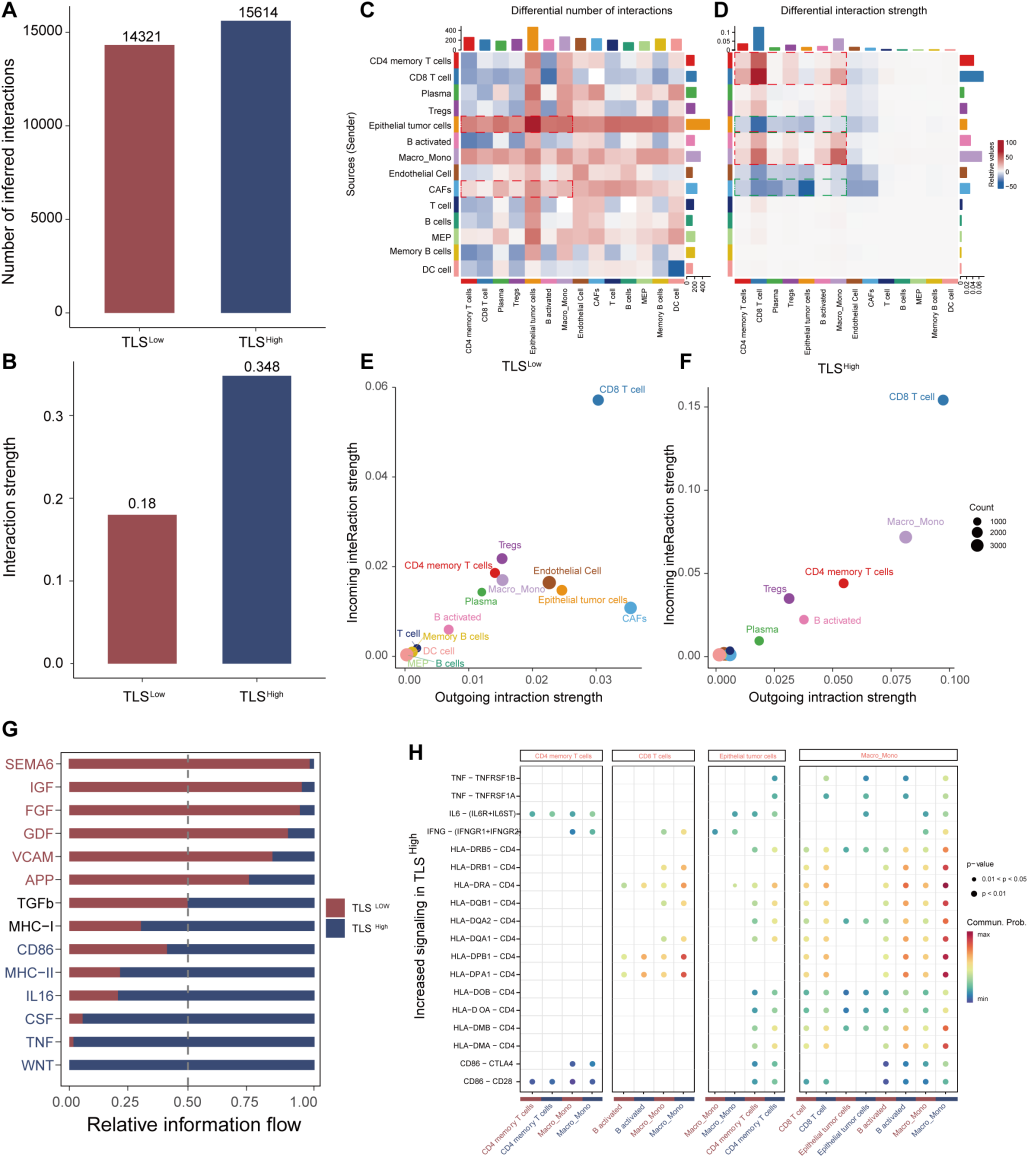
Analysis of cell communication between TLS high and low groups. Bar graphs of the total number of interactions **(A)** and interaction strength **(B)** of intercellular communication networks of TLS high and low groups. Heat map of the degree of change in the number of interactions **(C)** or interaction strength **(D)** of the high TLS group compared with the low group. Scatter plots of signal emission and reception of the low TLS group **(E)** and the high TLS group **(F, G)** Information flow diagram of the signal pathway. **(H)** Comparative scatter plots of signal transduction ligand-receptor pairs.

To further explore the potential mechanism by which TLS modulates TME in colorectal cancer, we performed a systematic cell communication analysis comparing TLS^High^ and TLS^Low^ groups using single-cell transcriptomic data. The results showed that both the number of cell-to-cell interactions and the overall signal transmission intensity were markedly higher than those in the TLS^High^ group (**Figures 6A, B**), suggesting that TLS may facilitate the remodeling of the immune microenvironment by enhancing immune communication and signal exchange between cells. Subsequent heat map analysis (**Figures 6C, D**) further highlighted the differences in the number and intensity of interactions between various cell subsets in the TLS^High^ and TLS^Low^ groups. In the TLS^High^ group, the interaction between immune cells was remarkably enhanced, especially the frequency and intensity of interactions between CD4 memory T cells, CD8 T cells, B activated, and Macro_Mono, indicating that a closer signal network was formed among immune cells in the context of TLS enrichment. Interestingly, the number of interactions among epithelial tumor cells, CAFs, and immune cells (such as CD4 memory T cells, Macro_Mono, activated B cells, etc.) also increased significantly within the TME of the TLS^high^ group, suggesting that tumor cells and matrix components may try to establish more connections with immune cells in an immune-active environment to regulate their own behavior. However, the signal transmission intensity of these interactions was weakened in the TLS^High^ group, suggesting that under high TLS, tumor cells and stromal cells may change from the original immunosuppression or escape state toward a more mild and even regulatory interaction mode that potentially facilitates local immune response. This observation further underscores the key regulatory role of TLS in shaping the anti-tumor immune microenvironment.

Systematic evaluation of signal transmission (outgoing) and reception (incoming) capabilities of cells in the TLS^High^ and TLS^Low^ groups revealed that CD4 memory T cells, CD8 T cells, and Macro_Mono in the TLS^High^ group exhibited bidirectional enhancement in signal activity, demonstrating strong transmission and reception capabilities simultaneously (**Figures 6E, F**). This suggests they are not only active information transmitters in the cell communication network but also may serve as important immune signal integration nodes. Activated B cells mainly act as signal transmitters, promoting immune activation. In contrast, epithelial tumor cells and CAFs showed significantly reduced signaling capability in the TLS^High^ group, suggesting a decline in their immune regulatory control, which may promote immune cell dominance and microenvironmental reprogramming.

Further analysis of the information flow distribution of signal pathways showed that the TLS^High^ group significantly enhanced the activity of multiple key signal pathways, including CD86, MHC-II, IL16, TNF, and WNT, which are widely involved in immune-related processes such as T cell activation, antigen presentation, and inflammation regulation. In contrast, the TLS^Low^ group was enriched for pathways such as SEMA6, FGF, GDF, and VCAM (**Figure 6G**), which are primarily related to tumor progression, immunosuppression, and angiogenesis, suggesting that TLS deficiency may contribute to immune escape or the establishment of a tumor-promoting environment. The bubble chart further identified ligand-receptor pairs that were specifically upregulated in the TLS^High^ group (**Figure 6H**), such as the self-feedback activation between CD4 memory T cells → CD4 memory T cells, and the enhancement of CD86–CTLA4 and CD86–CD28 pairing signals in Macro_Mono, indicating robust activation of T cell co-stimulatory signals. In addition, enhanced signal transduction from CD8 T cells and epithelial tumor cells to activated B cells, along with the upregulation of HLA-II class molecules in Macro_Mono and CD8 T cells, pointed to elevated antigen presentation and immune recognition capability in the TLS^High^ group.

In summary, the TLS structure may enhance the anti-tumor immune response by promoting the communication and coordination between immune cells, mitigating the immunosuppressive properties of tumor and stromal cells, and reshaping the signal network pattern within TME. These results support TLS as a potential immunotherapy target in improving the poor prognosis context, such as BRAF mutations.

### 3.7 Prediction of anticancer treatment response in CRC patients with BRAF mutations with TLS high and low prognostic scores

**Figure 7:**
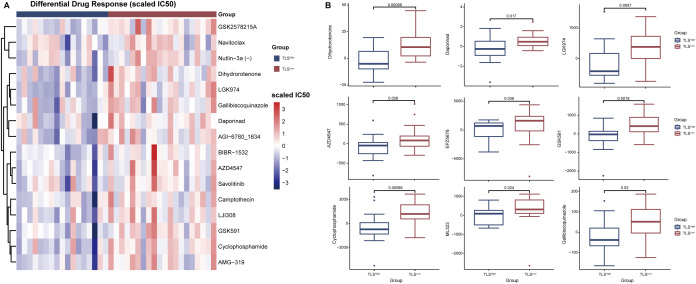
Analysis of drug sensitivity in TLS^High^ and TLS^Low^ groups in patients with BRAF mutations. **(A)** Heat map of drug-scaled IC50 in TLS^High^ group and TLS^Low^ group. **(B)** Box plot of drug lod (limit of detection) IC50 in TLS^High^ group and TLS^Low^ group.

We further evaluated the differences in anticancer drug response between TLS^Low^ and TLS^High^ groups harboring BRAF mutations. The results showed that the estimated IC50 values in the TLS^Low^ group were generally higher than those in the TLS^High^ group (**Figure 7A**), indicating stronger resistance to multiple drugs. Notably, the TLS^low^ group exhibited significantly higher IC50 values for both targeted therapy drugs (such as AZD4547, GSK591, EPZ5676) and chemotherapy drugs (such as Cyclophosphamide, Vinblastine), suggesting a reduced drug sensitivity associated with low TLS activity (**Figure 7B**). TLS is generally considered to play an active role in tumor immune response, and its loss or inadequate function may lead to increased resistance of tumor cells to chemotherapy and targeted drugs. Based on current data, TLS may enhance the efficacy of drugs by regulating the immune microenvironment, thereby reducing the drug resistance of CRCs driven by BRAF mutation.

## 4 Discussion

BRAF is a member of the RAF kinase family (a serine/threonine-protein kinase family), which transduces signals downstream of RAS via the mitogen-activated protein kinase (MAPK) pathway. About 200 *BRAF* mutations have been identified, occurring in both functional and non-functional regions. The most common and well-known *BRAF* mutation is V600E (35), which has been detected in various cancers, such as malignant melanoma (36, 37), papillary thyroid cancer (38, 39), and colorectal cancer (11). Current clinical RAF inhibitors suppress RAF activity and downstream ERK signaling selectively in cells expressing mutant BRAF. Two BRAF inhibitors, vemurafenib and dabrafenib, have been approved for the treatment of melanoma (40–43). However, due to the activation of parallel oncogenic pathways such as RAS-MAPK or PI3K/Akt/mTOR and other reasons, resistance to these agents frequently develops (44, 45). Patients with BRAF^V600E^-mutated CRC face a poor prognosis, with a median OS of 11 months and a lack of response to standard therapies. A phase I study in patients with metastatic CRC harboring the BRAF^V600E^ mutation demonstrated that the BRAF inhibitor (BRAFi) vemurafenib has no clinical benefit when given as monotherapy (44).

In recent years, increasing evidence has shown that the presence of TLS generally confers a positive prognostic value in most solid tumors (15, 21), such as lung cancer (46) and pancreatic cancer (47). Experimental studies have successfully induced the formation of TLS by local expression of TLS-associated cytokines or chemokines, enhancing anti-tumor immune responses (48). Notably, immune checkpoint blockade has also been shown to induce the formation of TLS in tumors (49). Our study revealed that while BRAF^MT^ CRC patients showed worse prognosis than BRAF^WT^ cases, stratification by intratumoral TLS abundance eliminated this survival disparity. Notably, BRAF^WT^ patients with low TLS (TLS^Low^) and BRAF^MT^ patients with high TLS (TLS^High^) demonstrated comparable OS, suggesting that robust TLS formation may overcome the adverse prognostic impact of BRAF mutations in CRC.

This study employed a multi-modal approach, integrating RNA-Seq, scRNA-Seq data, clinical outcomes, and pathological assessment to systematically explore the prognostic value and immunological significance of TLS in CRC. Our analyses revealed that TLS-associated gene expression profiles were not only remarkably altered in COAD tumor tissues compared to normal tissues, but also had significant potential in risk stratification and prognostic prediction.

Using unsupervised consensus cluster analysis, we identified two distinct TLS-related clusters associated with different overall survival outcomes. Patients in the TLS^High^ cluster had significantly longer survival than those in the TLS^Low^ cluster, supporting the view that TLS-rich tumors may promote more effective antitumor immunity. Based on this observation, we constructed a robust prognostic signature containing 10 key genes (*CCL19, CCL22, ICOS, IGHG1, JCHAIN, CD37, XBP1, FCMR, TNFRSF13C*, and *FCRLA*), using univariate and LASSO Cox regression. Among these, *CCL19* and *CCL22* are chemokines involved in lymphocyte recruitment and organization of the lymphoid microenvironment in TME. ICOS is a key co-stimulatory molecule for T cell activation, while *IGHG1, JCHAIN*, and *FCRLA* reflect B cell maturation and antibody production, which are hallmarks of active humoral immunity. Genes such as *CD37, XBP1*, and *FCMR* are involved in B cell receptor signaling, plasma cell differentiation, and immunoglobulin homeostasis, respectively. In addition, *TNFRSF13C* (also known as *BAFFR*) plays a key role in B cell survival and TLS maintenance. The model demonstrated consistent prognostic performance across multiple time points, with clear survival differences between TLS^High^ and TLS^Low^ groups.

In addition to these 10 key feature genes, we examined the broader set of 121 TLS-related genes. Among these, 110 genes showed significant differential expression between TLS^High^ and TLS^Low^ groups (**Supplementary Figure 5A**). Notably, these 10 feature genes were significantly enriched in several key pathways related to immune regulation, inflammation, and cellular stress responses, such as “the interleukin-10 signaling pathway”, “chemokine receptor and ligand binding”, “peptide ligand-receptor interaction”, “TNF receptor superfamily members mediating non-canonical NF-κB signaling”, “the unfolded protein response (UPR) “, and “the PI3K/AKT signaling pathway in cancer” (**Supplementary Figure 5B**, **Supplementary Table 6**).

These pathways suggest that the feature genes play a critical role in immune homeostasis, cell survival under stress conditions, and the resolution of inflammation, which is consistent with their prognostic value in our model. For example, XBP1 is a core regulator of the UPR pathway, and its activation reflects endoplasmic reticulum stress and immune regulation; meanwhile, TNFRSF13C and FCMR are involved in B cell survival and differentiation through non-canonical NF-κB signaling.

In contrast, enrichment analysis of the remaining 111 genes revealed that, although they were involved in a broader range of immune and metabolic pathways, many of these pathways (e.g., “integrin interactions”, “vitamin metabolism”, “platelet activation”) showed less direct association with disease prognosis and more reflected general immune or cellular functions (**Supplementary Figure 5C**, **Supplementary Table 7**).

Importantly, there were some overlapping pathways between the two gene groups, such as “chemokine signaling,” “interleukin signaling,” and “scavenger receptor binding,” suggesting that although many genes participate in immune-related processes, the 10 feature genes represent core components or regulators of these pathways, which may explain their stronger prognostic relevance.

Therefore, our selection strategy appears to have captured genes that are not only statistically significant but also biologically core to key immune-related pathways.

Importantly, our immunological profiles revealed significant differences between the two risk groups. The TLS^Low^ group was characterized by a “cold” immune phenotype with low stromal and immune scores and was enriched in immunosuppressive cells, such as Tregs and M0 macrophages. In contrast, the TLS^High^ group exhibited a more immunoreactive microenvironment, with higher infiltration of effector immune cells, including memory CD4^+^ T cells, dendritic cells, and neutrophils. These findings are consistent with previous studies suggesting that TLS functions as a local immune hub that recruits and activates immune cells within TME, thereby enhancing anti-tumor immune surveillance. Furthermore, TIDE analysis indicated that TLS^Low^ patients were more prone to immune evasion and exhibited a reduced MSI score, suggesting poor responsiveness to ICI treatment. Collectively, these results highlight the potential of TLS-based profiling not only for prognostic evaluation but also for predicting the efficacy of immunotherapies.

Notably, our study also explored the interaction between TLS and BRAF mutation status, a well-recognized adverse prognostic factor in COAD. Stratified survival analysis showed that TLS^High^ patients had significantly better survival outcomes than TLS^Low^ patients carrying BRAF mutations. These findings suggest that TLS may partially offset the pro-tumor effects of BRAF mutations, potentially by reshaping the immune environment to promote anti-tumor responses. Supporting this hypothesis, transcriptome analysis of CRC carrying BRAF mutations showed that complement pathways, inflammatory response genes, and epithelial junction integrity were all upregulated in TLS^High^ tumors.

Histopathological analysis of 200 clinical CRC specimens reinforced the association between BRAF mutation and TLS inhibition. Tumors harboring BRAF^MT^ had significantly fewer and less mature intratumoral TLS compared to BRAF^WT^ tumors, suggesting that BRAF mutations may actively suppress TLS formation or maturation. This is consistent with previous findings showing that inhibition of the MAPK pathway elevated the immune signatures (T cell, cytotoxic T cell, and phagocytes) in BRAF V600E mutation CRC (13). This provides new mechanistic insights into how oncogenic pathways modulate the tumor immune landscape to escape immune surveillance.

In conclusion, our findings highlight the dual prognostic and immunological relevance of TLS in COAD. TLS-based risk stratification not only provides prognostic value but also reflects key features of the tumor immune microenvironment, shedding light on immune escape mechanisms and therapeutic vulnerabilities. Moreover, TLS may mitigate BRAF-driven tumor progression, highlighting its potential as a promising therapeutic target or biomarker in the context of precision oncology.

However, the limitations of our study include that, firstly, most analyses were based on retrospective datasets. Secondly, as BRAF mutation and TLS high are a relatively rare combination, our results would need to be further validated using large-scale samples. Thirdly, the mechanistic association between TLS inhibition and BRAF signaling requires in-depth investigation in experimental models, and future studies integrating spatial transcriptomics, multiplex immunohistochemistry, and functional analysis should clarify the causal relationship between BRAF mutation and TLS formation/maturation while unraveling the interaction mechanisms among TLS, tumor cells, and immune components.

In conclusion, TLS-related gene signatures establish a novel framework for prognostic prediction and immunological stratification of COAD, particularly in patients with high-risk BRAF mutations. TLS profiling holds potential to refine immunotherapy strategies and to identify patient subgroups most likely to benefit from immune-enhancing interventions.

## Data Availability

The original contributions presented in the study are included in the article/**Supplementary Material**. Further inquiries can be directed to the corresponding authors.
