# Supplementary material for: A multi-omic analysis reveals a predictive value of tertiary lymphoid structures in improving the prognosis of colorectal cancer patients with BRAF mutation
Source: Front Immunol. 2025 Sep 1;16:1662573. doi: 10.3389/fimmu.2025.1662573 (PMC12434122; doi:10.3389/fimmu.2025.1662573)
Supplement: Supplementary file 1 [file DataSheet1.docx]

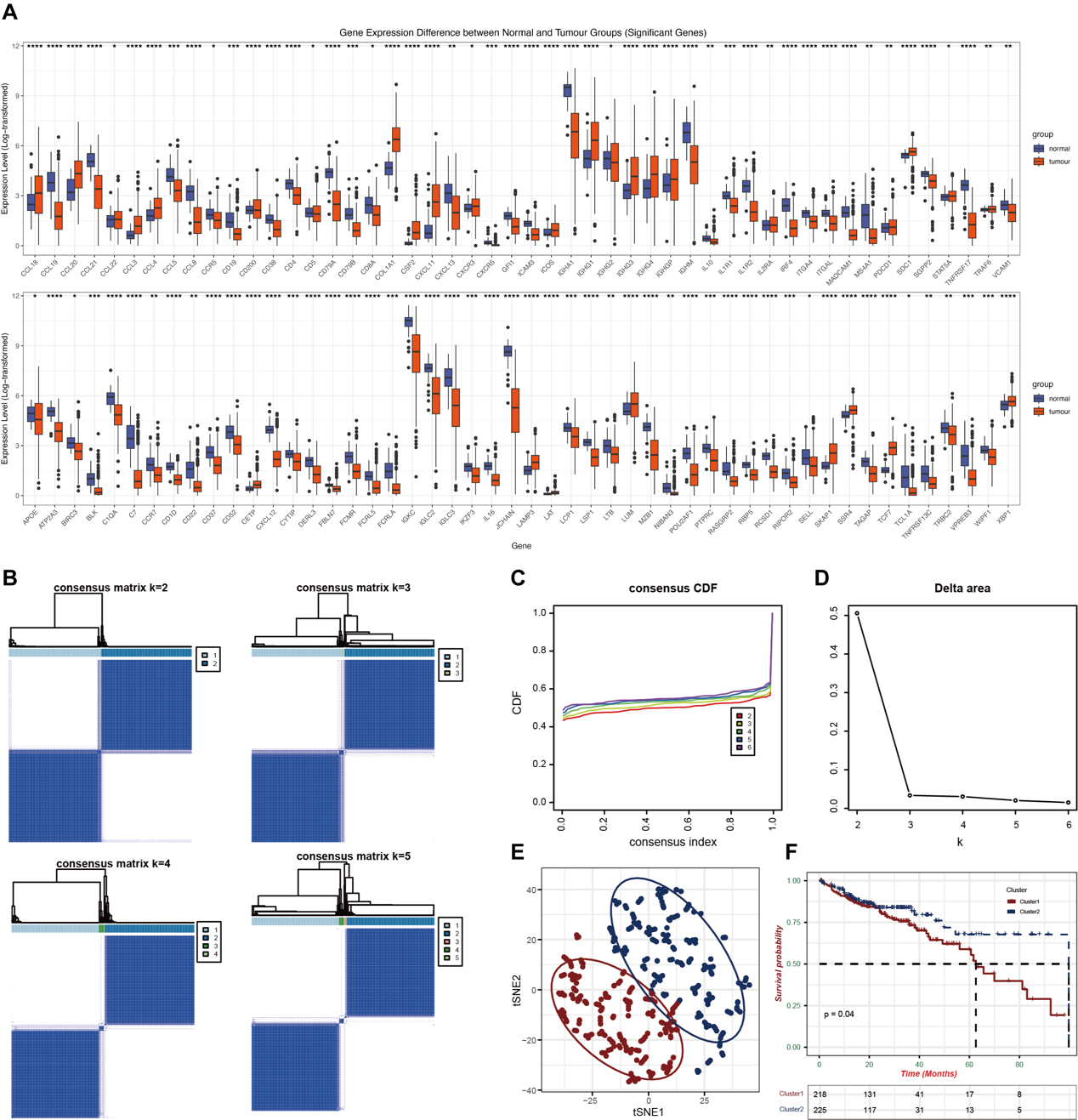


**Supplementary Figure 1.** Clustering based on TLS in colorectal cancer.

**(A)** Box plot of TLS-related gene sets in TCGA-COAD in tumor and adjacent tissues. (**B)** Consistent clustering matrix with K values ​​from 2 to 5. B. Sample distribution tracking with K values ​​from 2 to 6. **(C)** Relative change of area under the CDF curve. (**D)** Consistent clustering CDF curve with K values ​​from 2 to 6. (**E**) t-SNE dimensionality reduction analysis of two clusters. (**F**) Kaplan-Meier survival analysis of two clusters.


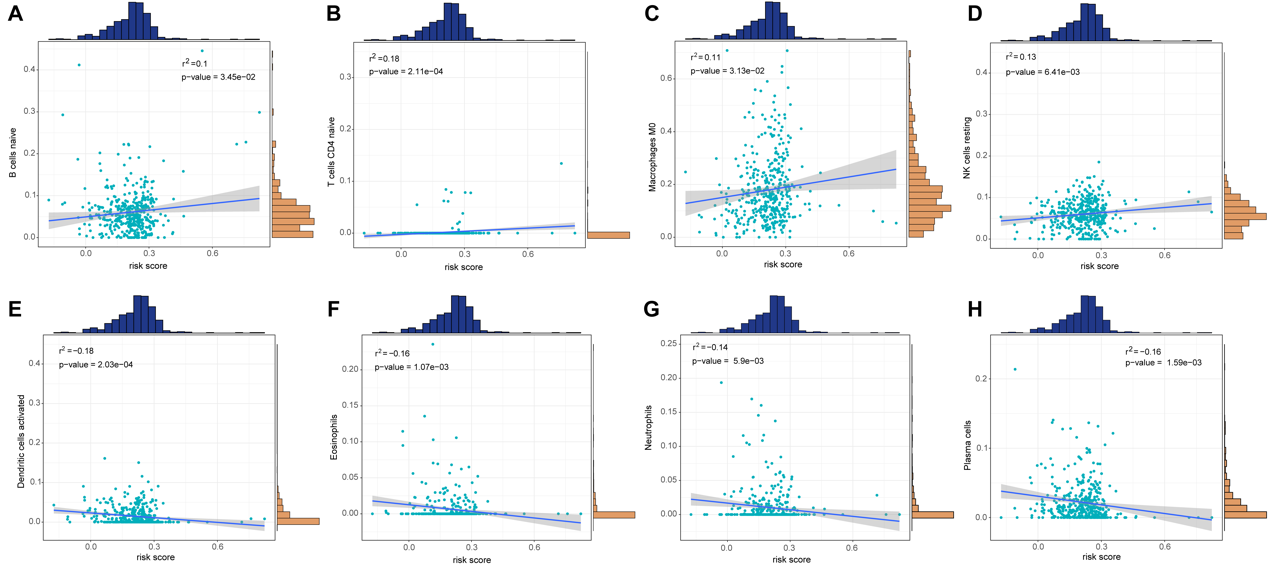


**Supplementary Figure 2.（A-H）** Correlation between the proportion of different immune cells and risk score.


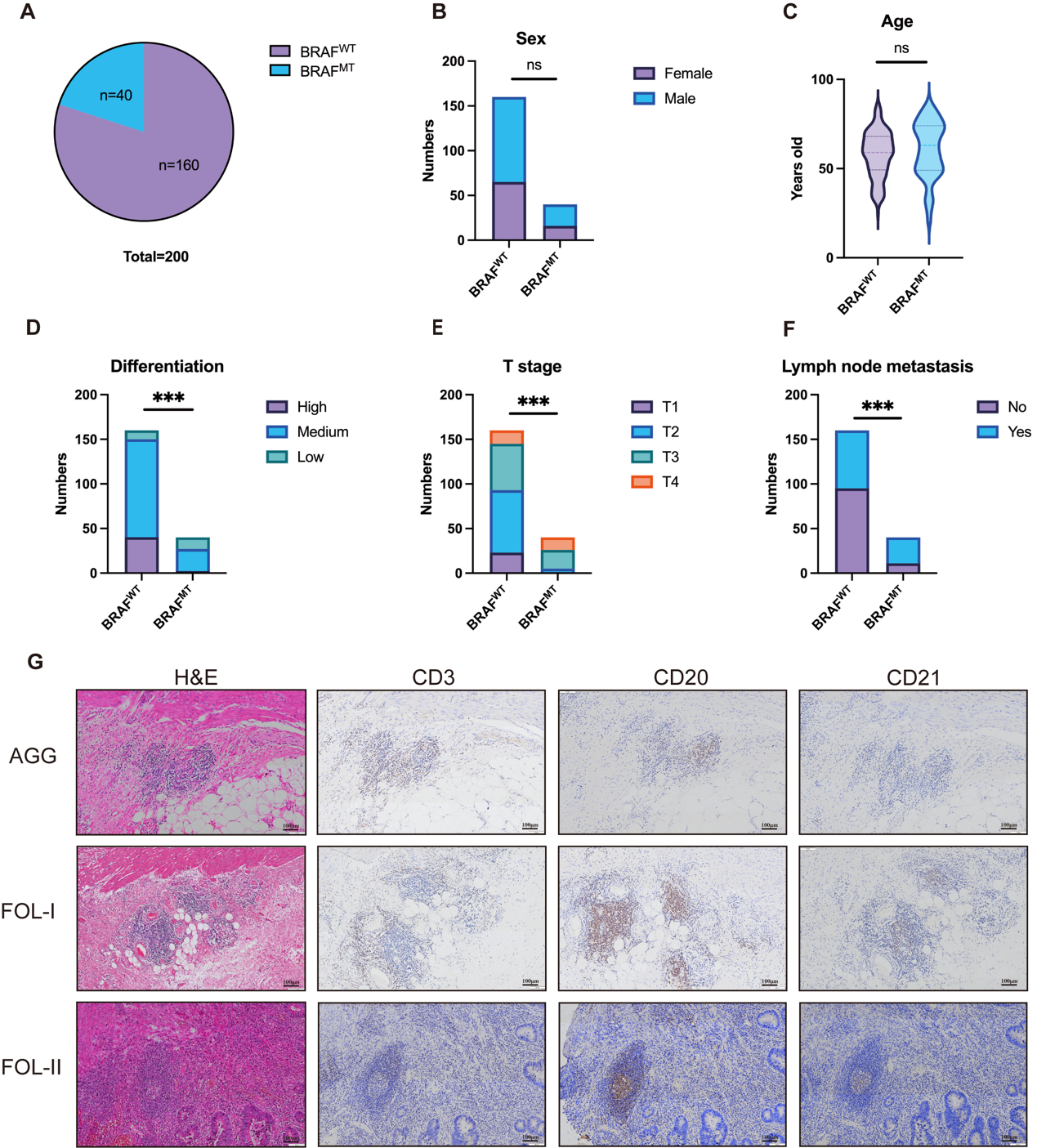


**Supplementary Figure 3.** Differences in clinicopathological and prognostic characteristics between BRAF^WT^ and BRAF^MT^ CRCs, and morphological characteristics of TLSs at different stages of maturity. **(A)** Two hundred CRCs were enrolled, among which 40 (20%) were BRAF^MT^, and 160 (80%) were BRAF^WT^. **(B-F)** Statistical analysis of sex, age, tumor differentiation, tumor stage, and lymph node metastasis between BRAF^WT^ and BRAF^MT^ patients, respectively. ns: no statistically significant difference; ***p<0.001. **(G)** AGG: CD3+, and CD20+ lymphocyte clusters lacking CD21; FOL-I: CD21 signal expression in dense CD20+ lymphocyte clusters; FOL-II: CD21 signal expression in dense CD20+ lymphocyte clusters. H&E shows a germinal center in the center of the follicle. Scale bar:100 um.


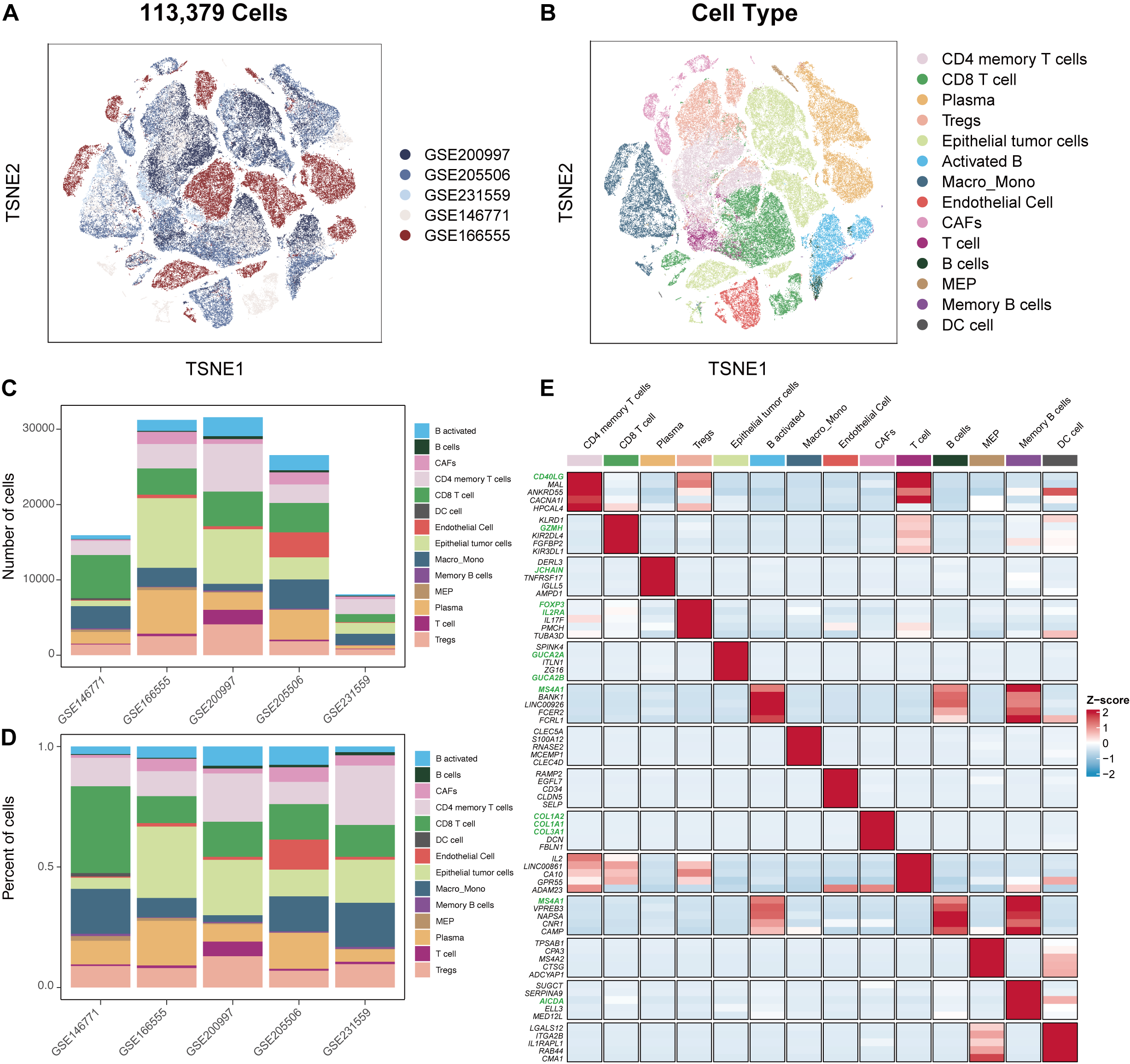


**Supplementary Figure 4.** Distribution characteristics of TLS in single-cell data of colorectal cancer. 5 scRNA-seq datasets, a total of 113,379 cells. **(A)** 14 types of cell types **(B)**. Cell quantity **(C)** and cell proportion **(D)** of 5 scRNA-seq datasets. **(E)** Top 5 feature genes of 14 types of cell subsets


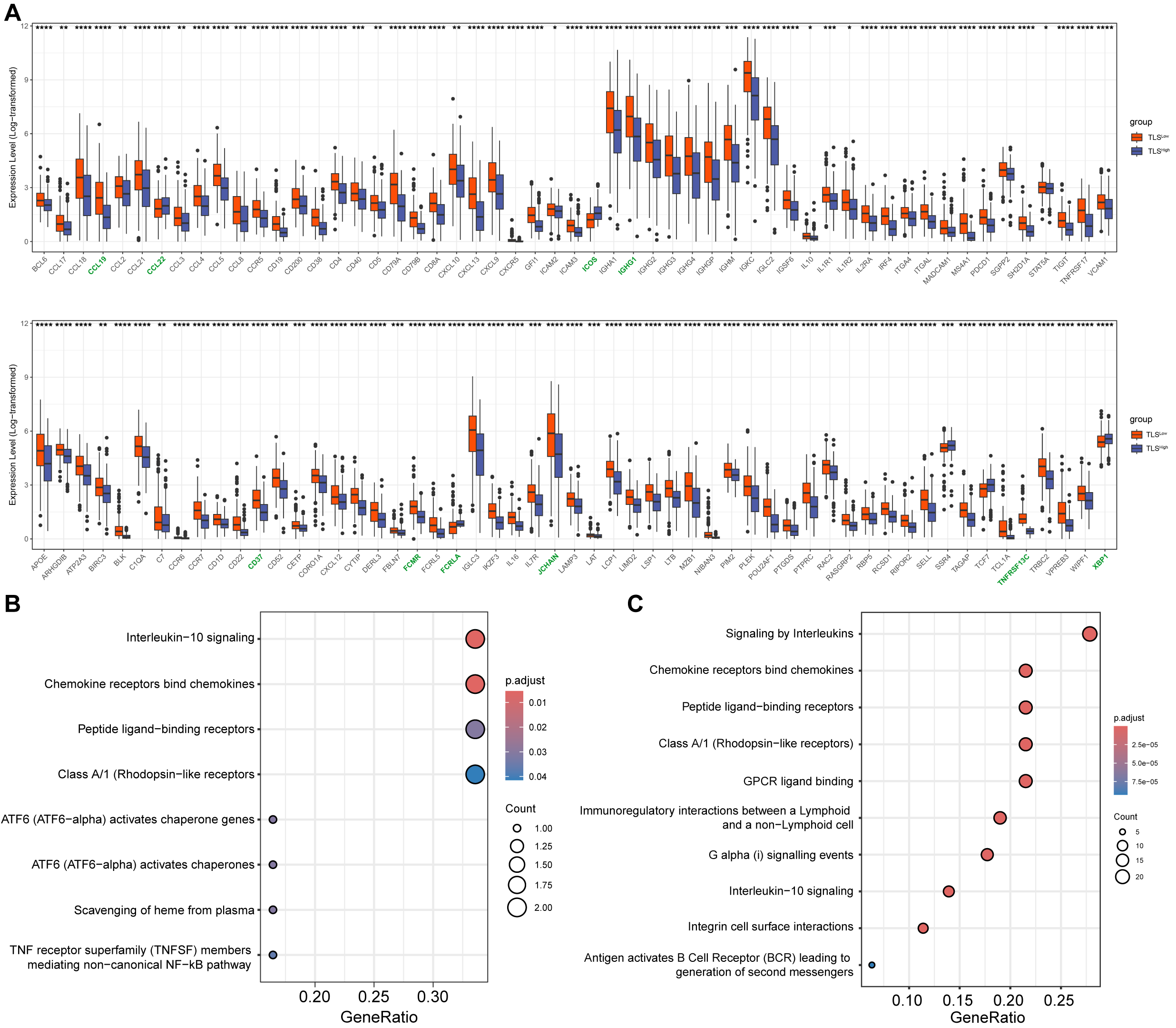


**Supplementary Figure 5.** Expression and pathway enrichment analysis based on TLS-related genes. **(A)** Box plot of TLS-related gene sets between high and low TLS groups in the TCGA-COAD cohort. **(B)** Dotplot of pathway enrichment analysis of 10 model genes. **(C)** Dotplot of pathway enrichment analysis of 111 non-model genes defined as TLS-related genes
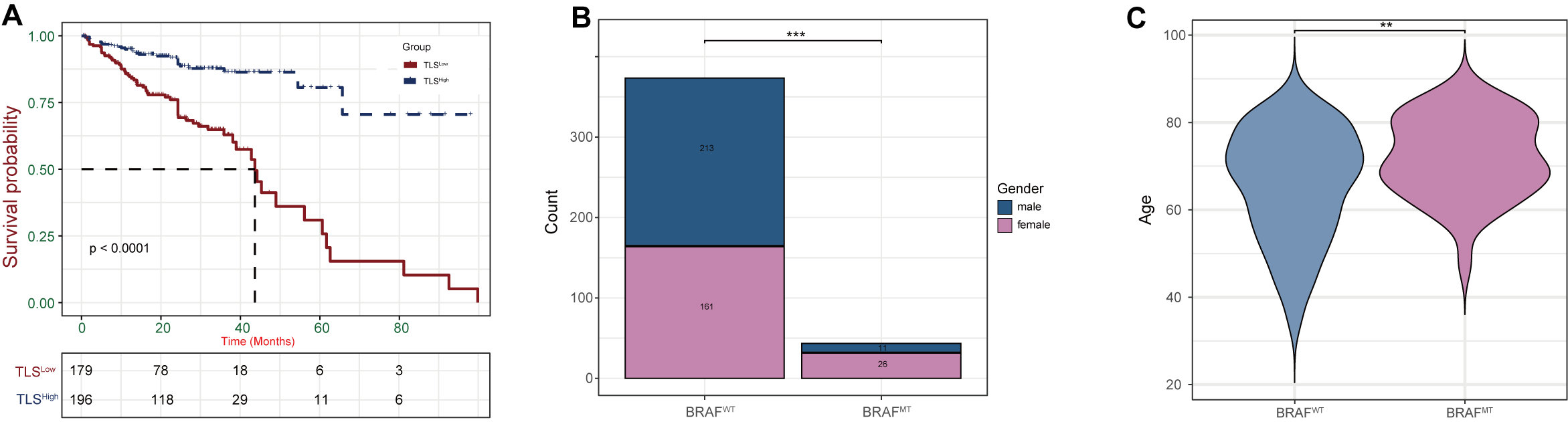


**Supplementary Figure 6.** Analysis of COAD patients. **(A)** Survival analysis of TLS groups within BRAF wild type samples**;** Statistical comparisons of gender **(B)** and age **(C)** distributions between BRAF^WT^ and BRAF^MT^ patients**.**

**Supplementary Table 1.** 121 TLS-related genes

| Gene | Gene | Gene | Gene | Gene | Gene |
| --- | --- | --- | --- | --- | --- |
| ICAM2 | CXCL11 | IL1R2 | IGHM | ARHGDIB | SKAP1 |
| ICAM3 | CD200 | IL10 | IGKC | BIRC3 | CETP |
| VCAM1 | ICOS | CCL20 | IGLC2 | CORO1A | EIF1AY |
| MADCAM1 | SGPP2 | IRF4 | IGLC3 | CYTIP | RBP5 |
| ITGAL | SH2D1A | TRAF6 | JCHAIN | LCP1 | FBLN7 |
| ITGA4 | TIGIT | STAT5A | CD52 | LIMD2 | CD22 |
| ITGAD | PDCD1 | TNFRSF17 | FCRL5 | LSP1 | CD37 |
| CCL19 | CD4 | CD19 | MZB1 | LTB | VPREB3 |
| CCL21 | CCR5 | CD79A | SSR4 | PLEK | FCMR |
| CXCL13 | CXCR3 | CD79B | XBP1 | POU2AF1 | TCF7 |
| CCL17 | CSF2 | CXCR5 | TRBC2 | PTPRC | TNFRSF13C |
| CCL22 | IGSF6 | BCL6 | IL7R | RCSD1 | BLK |
| CCL2 | IL2RA | CD8A | CXCL12 | SELL | FCRLA |
| CCL3 | CD38 | COL1A1 | LUM | TAGAP | IKZF3 |
| CCL4 | CD40 | IGHA1 | C1QA | WIPF1 | NIBAN3 |
| CCL5 | CD5 | IGHG1 | C7 | CCR6 | RASGRP2 |
| CCL8 | MS4A1 | IGHG2 | APOE | CCR7 | TCL1A |
| CCL18 | SDC1 | IGHG3 | PTGDS | LAMP3 | RAC2 |
| CXCL9 | GFI1 | IGHG4 | PIM2 | CD1D | ATP2A3 |
| CXCL10 | IL1R1 | IGHGP | DERL3 | LAT | IL16 |
| RIPOR2 |  |  |  |  |  |

**Supplementary Table 2.** GSEA enrichment analysis of the TLS^High^ group compared with the TLS^Low^ group in patients with BRAF mutation

| **Description** | **enrichmentScore** | **NES** | **p-value** | **p.adjust** | **q-values** |
| --- | --- | --- | --- | --- | --- |
| HALLMARK_ESTROGEN_  RESPONSE_LATE | 0.84 | 2.68 | 0.00 | 0.00 | 0.00 |
| HALLMARK_ESTROGEN_  RESPONSE_EARLY | 0.80 | 2.56 | 0.00 | 0.00 | 0.00 |
| HALLMARK_APICAL_J  UNCTION | 0.75 | 2.80 | 0.00 | 0.00 | 0.00 |
| HALLMARK_COMPLEMENT | 0.77 | 2.27 | 0.00 | 0.00 | 0.00 |
| HALLMARK_COAGULATION | 0.63 | 2.01 | 0.00 | 0.01 | 0.00 |
| HALLMARK_EPITHELIAL_  MESENCHYMAL_TRANSITION | 0.59 | 1.90 | 0.01 | 0.01 | 0.00 |
| HALLMARK_KRAS_SIGNALING_DN | 0.48 | 1.87 | 0.01 | 0.02 | 0.00 |
| HALLMARK_  INFLAMMATORY_RESPONSE | 0.59 | 1.76 | 0.03 | 0.03 | 0.00 |
| HALLMARK_KRAS_  SIGNALING_UP | -0.28 | -0.77 | 0.76 | 0.76 | 0.09 |

**Supplementary Table 3.** Correlation between Clinicopathologic Features and BRAF mutation in 200 CRC patients

| Clinical characteristics | Group | Total | BRAF | | P value |
| --- | --- | --- | --- | --- | --- |
|  |  |  | Wild type | Mutant |  |
|  |  |  | (n=160) | (n=40) |  |
| Gender | Female | 81 | 65 | 16 | 0.943 |
|  | Male | 119 | 95 | 24 |  |
| Age | <50 | 51 | 39 | 12 | 0.465 |
|  | ≥50 | 149 | 121 | 28 |  |
| Differentiation | High | 42 | 40 | 2 | <0.001 |
|  | Medium | 135 | 110 | 25 |  |
|  | Low | 23 | 10 | 13 |  |
| T stage | T1 | 24 | 23 | 1 | <0.001 |
|  | T2 | 74 | 80 | 4 |  |
|  | T3 | 73 | 52 | 21 |  |
|  | T4 | 29 | 15 | 14 |  |
| Lymph node metastasis | No | 106 | 95 | 11 | <0.001 |
|  | Yes | 94 | 65 | 29 |  |

**Supplementary Table 4.** Correlation of TLS location and BRAF mutation in 200 CRC patients

| TLS location | Grade | Count | BRAF V600E | | P value |
| --- | --- | --- | --- | --- | --- |
|  |  |  | Wild type | Mutant |  |
| Intra-tumor | 0 | 14 | 5 | 9 | <0.001 |
|  | 1 | 103 | 78 | 25 |  |
|  | 2 | 69 | 64 | 5 |  |
|  | 3 | 14 | 13 | 1 |  |
| Invasive margin | 0 | 35 | 26 | 9 | 0.083 |
|  | 1 | 134 | 104 | 30 |  |
|  | 2 | 27 | 26 | 1 |  |
|  | 3 | 4 | 4 | 0 |  |
| Peri-tumor | 0 | 80 | 68 | 12 | 0.087 |
|  | 1 | 114 | 89 | 25 |  |
|  | 2 | 6 | 3 | 3 |  |

**Supplementary Table 5.** Correlation of intra-tumor TLS maturity and BRAF mutation in 200 CRC patients

| Intra-tumor maturity | Grade | Count | BRAF V600E | | P value |
| --- | --- | --- | --- | --- | --- |
|  |  |  | Wild type | Mutant |  |
| AGG | 0 | 28 | 16 | 12 | 0.002 |
|  | 1 | 157 | 133 | 24 |  |
|  | 2 | 10 | 6 | 4 |  |
|  | 3 | 5 | 5 | 0 |  |
| FOL-I | 0 | 47 | 22 | 25 | <0.001 |
|  | 1 | 149 | 134 | 15 |  |
|  | 2 | 3 | 3 | 0 |  |
|  | 3 | 1 | 1 | 0 |  |
| FOL-II | 0 | 180 | 144 | 36 | 1.000 |
|  | 1 | 20 | 16 | 4 |  |

**Supplementary Table 6.** Enrichment analysis of 10 feature genes

**Supplementary Table 7.** Enrichment analysis of the remaining 111 TLS related genes

**Supplementary Table 8.** Differentially expressed genes in CD8 T cells in TLS TLS^High^ and TLS^Low^ tumors

**Supplementary Table 9.** Differentially expressed genes in CD4 memory T cells in TLS^High^ and TLS^Low^ tumors.

**Supplementary Table 10.** Comparison of Drug Sensitivity Between TLS^High^ and TLS^Low^ Subgroups in BRAF-Mutant Colorectal Cancer

**Supplementary Table 11.** Individual Log(IC50) Values for Each Drug in BRAF-Mutant Colorectal Cancer Samples
